# Salivary pH influence on mechanical and surface properties of high-impact acrylic denture resin

**DOI:** 10.6026/973206300214580

**Published:** 2025-12-15

**Authors:** Neelarapu Sanjana Supriya, Sujesh M, Ravi Kumar C, Rajanikanth A.V, Harilal G, Kavitha Ch

**Affiliations:** 1 Department of Prosthodontics and crown and bridge, Mamata Dental College and Hospital, Khammam, Telangana, India

**Keywords:** Denture base resins, polymethyl methacrylate, flexural strength, surface roughness, salivary ph, *in vitro* study

## Abstract

Polymethyl methacrylate (PMMA) has been widely used as a denture foundation material even though it does not have enough flexural strength. Hence, the flexural strength of heat-cured PMMA has been tested by incorporating polyethylene and polypropylene fibers in an *in vitro* experiment. The experiment on 120 controlled specimens was carried out via a three-point bending test. Results showed significant improvement with both fibers, with polypropylene (169.61 ± 16.30 MPa) providing superior reinforcement compared to polyethylene (129.29 ± 5.00 MPa) and control (99.91 ± 3.79 MPa) (p < 0.001). Thus, we show polypropylene fiber reinforcement as an effective approach to enhance the mechanical performance and longevity of PMMA dentures.

## Background:

The polymethyl methacrylate (PMMA) denture bases have more than 80 years of history due to the low cost of production, biocompatibility, ease of working with, and aesthetic appearance of the material [1]. Traditional heat-polymerized PMMA has been in prevalence although it possesses certain inherent flaws that could lead to catastrophic fractures of the individual healer in the event of prosthesis use or accidental falls [2]. These disadvantages involve the poor impact and flexural strength. Midline fractures of maxillary dentures are a common clinical problem, often attributed to fatigue failure from cyclic loading during mastication [3]. To address these shortcomings, high-impact acrylic resins were developed. These materials are typically PMMA copolymers reinforced with rubber particles, such as butadiene-styrene, which are grafted into the polymer matrix. The rubber phase acts to absorb and dissipate energy, thereby interrupting crack propagation and significantly improving the material's impact strength and fracture toughness [4]. While these materials offer enhanced durability, their long-term performance is intrinsically linked to their stability within the dynamic oral environment. The oral cavity is a complex ecosystem where dental prostheses are constantly exposed to saliva, thermal fluctuations, and mechanical loading. The makeup and pH of saliva might vary greatly, yet it plays a crucial role in dental health maintenance. Resting saliva typically has a pH between 6.7 and 7.4, although it may become acidic (pH < 5.5) for a number of reasons [5]. These include the consumption of acidic foods and beverages, fermentation of carbohydrates by oral microflora, and systemic conditions such as gastroesophageal reflux disease (GERD) or bulimia [6]. Patients with xerostomia also exhibit a lower salivary pH due to reduced buffering capacity, further increasing the risk of chemical degradation of restorative materials [7]. An acidic environment can accelerate the degradation of polymeric materials like PMMA. The ingress of water and acidic ions into the polymer matrix can act as a plasticizer, disrupting the secondary intermolecular bonds between polymer chains and reducing the material's mechanical properties, such as flexural strength and hardness [8]. Furthermore, chemical erosion of the resin surface can increase surface roughness. A rougher surface is more susceptible to staining, plaque accumulation, and microbial colonization, potentially leading to denture stomatitis and poor oral hygiene [9]. While several studies have investigated the properties of high-impact resins, and others have explored the effects of various solutions on PMMA, there is a lack of direct comparative data on how acidic salivary conditions affect the latest generation of commercially available high-impact materials. Therefore, it is of interest to investigate the effects of acidic and neutral artificial saliva on two different high-impact acrylic denture base resins' surface roughness and flexural strength.

## Materials and Methods:

## Study design and sample preparation:

We conducted this work in a controlled *in vitro* setting. Eighty rectangular specimens were made in accordance with the ASTM D790 standard measurements, which are 65 mm in length, 10 mm in width, and 3 mm in thickness. Standardized molds were made using a master stainless steel die of specified specifications. Two commercially available high-impact, heat-polymerized acrylic resins were selected: Trevalon HI (Dentsply Sirona, UK) and Acryl HI (Asian Acrylates, India). For each material, 40 specimens were prepared. Dentists used a dental flask to embed the master die in Type III dental stone (Kalstone, Kalabhai Karson Pvt. Ltd., India). To make room for the mold, the flask was opened after the stone had set, and the die was taken out. Once the separating material (Cold Mould Seal, DPI, India) had dried, it was applied to the surfaces of the molds. Mixing the polymer powder and monomer liquid for each resin according to the manufacturer's recommendations (2.4:1 by weight) was done before packing the mold with dough. Excess material was removed during trial closures using a hydraulic press (P400, Sirio Dental, Italy). At 3500 psi, the flasks were clamped and the final closure was done. We used a temperature-controlled water bath (Acrylizer C-73A, Confident, India) to conduct the polymerization process at 74°C for 2 hours, followed by 1 hour of terminal boiling at 100°C. Defrosting was performed when the flasks had cooled to room temperature after polymerization. The retrieved specimens were carefully finished using tungsten carbide burs to remove flash, followed by sequential wet sanding with 120-grit and 240-grit silicon carbide paper to ensure standardized dimensions and smooth surfaces without altering the test surfaces.

## Grouping and immersion protocol:

The 80 specimens were divided into four groups (n=20 per group):

[1] Group 1A: Trevalon HI specimens immersed in artificial saliva at pH 7.0 (Control).

[2] Group 2A: Trevalon HI specimens immersed in artificial saliva at pH 4.0 (Experimental).

[3] Group 1B: Acryl HI specimens immersed in artificial saliva at pH 7.0 (Control).

[4] Group 2B: Acryl HI specimens immersed in artificial saliva at pH 4.0 (Experimental).

The artificial saliva was prepared in-house with a composition of NaCl (0.4 g/L), KCl (0.4 g/L), CaCl_2_.2H_2_O (0.795 g/L), NaH_2_PO_4_.H_2_O (0.690 g/L), and Na_2_S.9H_2_O (0.005 g/L)." We made sure the neutral solution had a pH of 7.0. A pH of 4.0 was achieved in the acidic solution by adding 2% hydrochloric acid (HCl). For 30 days, the specimens were kept in an incubator at 37°C in sealed containers with their corresponding solutions. To keep the pH stable and avoid microbiological contamination, the solutions were changed every 48 hours.

## Flexural strength testing:

After the 30-day immersion period, flexural strength testing was performed on 10 specimens from each group. An instrument for conducting three-point bending tests was a universal testing machine manufactured in Japan by Shimadzu Autograph AG-IS. Two 50 mm-long supports were used to hold each specimen in place. The specimen was subjected to a compressive force (F) in the middle at a crosshead speed of 1 mm/min until fracture was detected. The software on the machine recorded the maximum load at fracture. S = 3FL / 2bd^2^, where 'b' is the specimen's breadth and 'd' is its thickness, was used to compute the flexural strength (S) in megapascals (MPa).

## Surface roughness evaluation:

To measure surface roughness, researchers utilized the other ten specimens from each set. We utilized a contact profilometer made in Japan (Mitutoyo Surftest SJ-210) that has a diamond stylus with a tip radius of 5 µm. The surface of each specimen was scanned three times in parallel, using a 4 mm traverse length and a 0.8 mm cutoff value. For every trace, the mean roughness (Ra) in micrometers (µm) was noted, and each specimen's final Ra value was computed by averaging the three measurements.

## Results:

For each of the four categories, Table 1 (see PDF) displays the average and standard deviation of flexural strength. The flexural strength that was measured for Group 1A (Trevalon HI, pH 7) was 129.03 ± 8.42 MPa, whereas the one for Group 2B (Acryl HI, pH 4) was 102.39 ± 5.88 MPa, the lowest. The results of the one-way ANOVA showed that there was a significant variation in flexural strength amongst the four groups (F (3, 36) = 21.45, p < 0.001). The results of Tukey's post-hoc analysis (Table 3 - see PDF) demonstrated that dipping Trevalon HI and Acryl HI into acidic saliva (pH 4) significantly decreased their flexural strengths (p < 0.001) in comparison to their control groups at pH 7. Also, at neutral pH (p = 0.005) and acidic pH (p = 0.041), Trevalon HI showed much greater flexural strength than Acryl HI. In Table 2 (see PDF), you can see the average values of surface roughness, or Ra. With an average Ra of 0.41 ± 0.04 µm, Group 1A (Trevalon HI, pH 7) had the smoothest surface. With an average Ra of 0.75 ± 0.06 µm, Group 2B (Acryl HI, pH 4) had the roughest surface. A significantly significant difference in surface roughness was shown by the ANOVA findings (F(3, 36) = 35.12, p < 0.001) across all groups. According to post-hoc comparisons (Table 3 - see PDF), surface roughness was found to be considerably higher in the acidic immersion condition compared to the neutral pH condition for both Trevalon HI (p < 0.001) and Acryl HI (p < 0.001). Trevalon HI also showed a much smoother surface compared to Acryl HI at pH 7 (p < 0.001) and pH 4 (p < 0.001).

## Discussion:

This *in vitro* research shows that both of the high-impact acrylic denture base resins examined had their flexural strength and surface integrity drastically reduced when exposed to acidic conditions. This supports the theory that the acidity or alkalinity of the mouth's environment is a major factor in how long polymeric dental prostheses last. The hydrolytic breakdown and plasticization of the PMMA matrix are responsible for the observed loss in flexural strength following immersion in pH 4 artificial saliva. PMMA is a polar polymer and susceptible to water sorption. When immersed, water molecules penetrate the polymer network, occupying space between the polymer chains and weakening the intermolecular van der Waals forces. This plasticizing effect reduces the material's stiffness and strength [10]. An acidic medium can exacerbate this process by catalyzing the hydrolysis of the ester groups in the PMMA chains, leading to chain scission and further compromising the material's mechanical properties [8, 11]. The results align with research by de Sahin *et al.* who also reported a decrease in the fracture resistance of acrylic resins exposed to a low pH environment [12]. The increase in surface roughness following exposure to the acidic solution is likely a result of surface erosion. The acidic attack can preferentially degrade the polymer matrix, potentially exposing the embedded rubber reinforcing particles or creating microscopic pits and fissures on the surface [13]. This chemical degradation alters the surface topography, leading to a higher Ra value. The clinical implications of increased surface roughness are significant. A rougher surface provides more retention sites for microorganisms, facilitating the formation of dental plaque and biofilm. As a result, you may have foul smells, discoloration of the prosthesis, and an increased likelihood of denture-induced stomatitis, an inflammatory disorder that affects many people who wear dentures [9, 14]. In this study, Trevalon HI consistently outperformed Acryl HI in both flexural strength and surface smoothness under both neutral and acidic conditions. The superior performance of Trevalon HI may be related to differences in its chemical composition, such as the type and concentration of the rubber reinforcement (butadiene-styrene), the degree of cross-linking, and the size and distribution of the polymer beads. Although manufacturers seldom provide their materials' precise proprietary composition, changes in these areas are known to have a major impact on the resin's ultimate mechanical and physical characteristics [4, 15]. The lower surface roughness of Trevalon HI might suggest a more homogenous and less porous polymer matrix that is more resistant to chemical erosion. The limitations of this study must be acknowledged. This *in vitro* investigation cannot fully replicate the complex dynamics of the oral environment, which involves fluctuating temperatures, variable masticatory forces, and the presence of enzymes and microorganisms. The use of a static immersion model and a single acidic agent (HCl) simplifies the *in vivo* situation. Future research could incorporate dynamic loading cycles to simulate mastication; use organic acids commonly found in the diet, and evaluate other critical properties like impact strength, color stability, and wear resistance over longer periods. Nevertheless, the results provide valuable insights for clinicians. For patients with known risk factors for an acidic oral environment, such as GERD, a high-sugar diet, or hyposalivation, the selection of a more chemically stable and durable denture base material is paramount. The findings suggest that not all high-impact resins perform equally, and choosing a material with proven resistance to acidic degradation, such as Trevalon HI in this study, could contribute to the longevity and clinical success of the prosthesis.

## Conclusion:

We show that high-impact denture base resins lose a lot of their flexural strength and surface smoothness when exposed to acidic oral conditions. Trevalon HI outperformed Acryl HI under both neutral and acidic conditions. Careful material selection is essential to ensure the long-term durability of dentures, particularly in patients predisposed to low salivary pH.
